# Aortic valve imaging using ^18^F-sodium fluoride: impact of triple motion correction

**DOI:** 10.1186/s40658-022-00433-7

**Published:** 2022-01-29

**Authors:** Martin Lyngby Lassen, Evangelos Tzolos, Daniele Massera, Sebastien Cadet, Rong Bing, Jacek Kwiecinski, Damini Dey, Daniel S. Berman, Marc R. Dweck, David E. Newby, Piotr J. Slomka

**Affiliations:** 1 Department of Medicine (Division of Artificial Intelligence in Medicine), Cedars-Sinai Medical Center, 8700 Beverly Blvd Ste. Metro 203, Los Angeles, CA 90048 USA; 2 Department of Clinical Physiology, Nuclear Medicine and PET and Cluster for Molecular Imaging, Department of Biomedical Sciences, Rigshospitalet and University of Copenhagen, Copenhagen, Denmark; 3 Department of Imaging, Cedars-Sinai Medical Center, 8700 Beverly Blvd Ste. Metro 203, Los Angeles, CA 90048 USA; 4 British Heart Foundation Centre for Cardiovascular Science, Clinical Research Imaging Centre, Edinburgh Heart Centre, University of Edinburgh, Edinburgh, UK; 5 Leon H. Charney Division of Cardiology, New York University School of Medicine, New York, NY USA; 6 Department of Interventional Cardiology and Angiology, Institute of Cardiology, Warsaw, Poland; 7 Department of Biomedical Sciences, Cedars-Sinai Medical Center, 8700 Beverly Blvd Ste. Metro 203, Los Angeles, CA 90048 USA

**Keywords:** Motion correction, PET/CT, Cardiac PET, ^18^F-sodium fluoride, Aortic valve imaging

## Abstract

**Background:**

Current ^18^F-NaF assessments of aortic valve microcalcification using ^18^F-NaF PET/CT are based on evaluations of end-diastolic or cardiac motion-corrected (ECG-MC) images, which are affected by both patient and respiratory motion. We aimed to test the impact of employing a triple motion correction technique (3 × MC), including cardiorespiratory and gross patient motion, on quantitative and qualitative measurements.

**Materials and methods:**

Fourteen patients with aortic stenosis underwent two repeat 30-min PET aortic valve scans within (29 ± 24) days. We considered three different image reconstruction protocols; an end-diastolic reconstruction protocol (standard) utilizing 25% of the acquired data, an ECG-gated (four ECG gates) reconstruction (ECG-MC), and a triple motion-corrected (3 × MC) dataset which corrects for both cardiorespiratory and patient motion. All datasets were compared to aortic valve calcification scores (AVCS), using the Agatston method, obtained from CT scans using correlation plots. We report SUV_max_ values measured in the aortic valve and maximum target-to-background ratios (TBR_max_) values after correcting for blood pool activity.

**Results:**

Compared to standard and ECG-MC reconstructions, increases in both SUV_max_ and TBR_max_ were observed following 3 × MC (SUV_max_: Standard = 2.8 ± 0.7, ECG-MC = 2.6 ± 0.6, and 3 × MC = 3.3 ± 0.9; TBR_max_: Standard = 2.7 ± 0.7, ECG-MC = 2.5 ± 0.6, and 3 × MC = 3.3 ± 1.2, all *p* values ≤ 0.05). 3 × MC had improved correlations (R^2^ value) to the AVCS when compared to the standard methods (SUV_max_: Standard = 0.10, ECG-MC = 0.10, and 3 × MC = 0.20; TBR_max_: Standard = 0.20, ECG-MC = 0.28, and 3 × MC = 0.46).

**Conclusion:**

3 × MC improves the correlation between the AVCS and SUV_max_ and TBR_max_ and should be considered in PET studies of aortic valves using ^18^F-NaF.

**Supplementary Information:**

The online version contains supplementary material available at 10.1186/s40658-022-00433-7.

## Background

Positron emission tomography (PET) utilizing ^18^F-sodium fluoride (^18^F-NaF) combined with computed tomography angiography (CTA) permits identification of microcalcification activity and progression of disease in aortic valves [1–6]. Moreover, ^18^F-NaF PET is currently being used as an endpoint in several ongoing clinical trials assessing the efficacy of novel therapies in aortic stenosis [7–9].

Cardiac ^18^F-NaF PET imaging is affected by motion (cardiac and respiratory [cardiorespiratory] and patient) and by technical challenges associated with the current acquisition protocols for ^18^F-NaF PET, which can last for up to 30 min. The long imaging protocols were initially designed to ensure sufficient count statistics to obtain images of diagnostic quality [10]. Unfortunately, patient motion during imaging protocols with long acquisition times degrades the image quality of the scans [10] and consequently reduces the quantitative accuracy and the test–retest repeatability [11, 12]. In addition, variations in the tracer injection-to-scan delays affect the quantitative assessment of the lesions with ^18^F-NaF PET [13–15]. While the impact of these factors is of keen interest in studies of aortic valve microcalcification, to date, it has been only evaluated in studies of coronary plaques [10–15]. Best possible image quality and spatial resolution are of critical importance in studies of microcalcification in native valves to understand the pathophysiology of aortic stenosis, and in studies of bioprosthetic valves where accurate localization of ^18^F-NaF uptake is essential.

To this end, we aimed to test the hypothesis that correcting for cardiorespiratory and patient motion (3 × MC) will lead to improved image quality (signal-to-noise ratio [SNR]) [10], and increased semi-quantitative measurements (maximum standardized uptake values [SUV_max_] and maximum target-to-background ratios [TBR_max_]) [11] in studies of microcalcification activity in the aortic valve.

## Methods

### Study population

Fourteen patients with aortic stenosis underwent repeated ^18^F-NaF aortic valve PET/CT examinations with the two occasions 4 weeks apart as a part of the ongoing study investigating the effect of drugs used to treat osteoporosis on the progression of calcific aortic stenosis (SALTIRE II) [7]. Of note, only the two baseline scans (scan 1 and scan 2) of the SALTIRE II study were evaluated in this study; thus, no disease progression was expected between the two scans. These scans were obtained originally to check the test–retest variability of the entire imaging procedure [10, 16].

This study was approved by the Scottish Research Ethics Committee and the Medicines and Healthcare Products Regulatory Authority of the United Kingdom, and the study was performed in accordance with the declaration of Helsinki. All participants signed written informed consent.

### Imaging protocol

*PET/CT* All patients underwent 30-min listmode PET-emission scans one hour following ^18^F-NaF injection (target injection 125 MBq). All scans were acquired on a 128-slice Biograph mCT system (Siemens Healthineers, Knoxville, USA). All patients had a low-dose CT scan for attenuation correction purposes prior to the PET acquisitions (120 kV, 50 mAs, 3-mm slice thickness). Three ECG leads were used for the detection of cardiac motion (ECG gating). No additional external markers for tracking patient or respiratory motion were employed.

*CT angiography* For anatomical identification of the aortic valves, all patients had a cardiac CT angiogram (CTA) for each scan session immediately after emission scanning. All CTA images were acquired using prospective gating, a 330-ms rotation time, and a body-mass index (BMI)-dependent voltage (BMI < 25 kg/m^2^, 100 kV, BMI ≥ 25 kg/m^2^, 120 kV). All patients had beta-blockers administered, either orally or intravenously to achieve a target heart rate of < 60 /min. Iodinated contrast was administered in bolus form (400 mg/mL) with an injection rate of 5–6 mL/s after determining the appropriate trigger delay, defined as a test-bolus of 20 mL of contrast material.

*Calcium scoring* Aortic valve CT calcium scoring was acquired for all patients to estimate the calcium scores for the repeat PET/CT scans and used for primary outcome of the study. A non-contrast ECG-gated CT was performed at each visit using the same scanner, electrocardiogram gating, and a standardized protocol (120 kV CARE Dose4D [Siemens], 3-mm slice thickness, spiral acquisition, 70% R-R interval, inspiratory breath-hold). CT calcium scoring was performed by an experienced operator using dedicated software (Vitrea Advanced; Toshiba Systems) on axial views, with care taken to exclude calcium originating from the ascending aorta, left ventricular outflow tract, and coronary arteries. The calcium score was recorded in Agatston units.

### Motion detection and image reconstruction

**Fig. 1:**
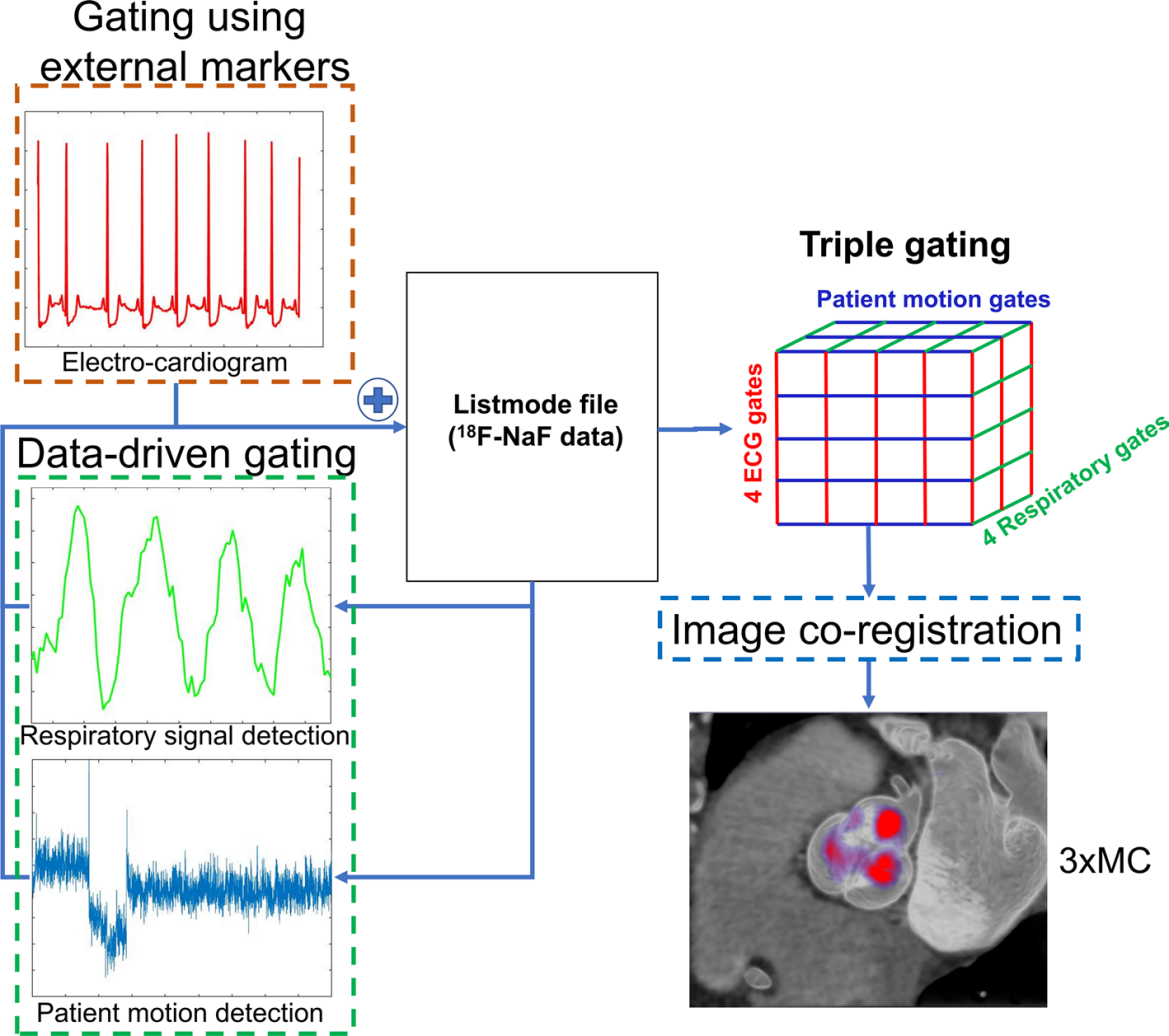
Overall scheme for simultaneous cardiorespiratory and gross patient motion detection and correction of ^18^F-NaF aortic valve PET scans. A fixed number of respiratory and electrocardiogram (ECG) gates (4 each) were used. The number of patient repositioning gates depended on the number of repositioning events the patient had during the acquisition. A 3D mesh of gated reconstructions were obtained, which were registered to generate the 3 × MC image set. 3 × MC = triple motion-corrected

Three different image reconstructions were evaluated in this study; (1) an end-diastolic image reconstruction employing 25% of the counts (Standard) [17] a cardiac motion-corrected dataset (ECG-MC) reconstruction employing four cardiac phases [10], and a triple motion-corrected (3 × MC) dataset employing 16 cardiorespiratory phases per each patient position phase as determined from the listmode. Thus, gross patient motion had variable number of phases. The 3 × MC reconstructions were obtained using a previously described gating protocol (Fig. 1) [12]. In brief, information on cardiac contraction was obtained using ECG gating. Respiratory and patient motion was detected retrospectively using data-driven gating. In brief, the data-driven gating employed a center-of-mass-based analysis of single-slice-rebinned sinograms (3D) obtained for every 200 ms [11, 18]. Information on the respiratory motion was extracted from the diaphragm region only using segmentation on the diaphragm area from the co-registered low-dose CT attenuation correction map, whereas gross body motion was detected by evaluating the whole PET field-of-view (longitudinal direction) as in work by Büther et al. [19] for respiratory motion detection. The motion detection was obtained using four time-of-flight bins covering the central part of the PET system (transaxial direction). For automatic extraction of patient repositioning events, the center-of-mass signal was filtered with a band-stop filter (0.2–0.5 Hz, equivocal to 12–30 respiratory cycles per minute) and a moving-average filter (3 s) to remove stochastic noise in the signal. Patient repositioning events were defined as changes in the center-of-mass baseline (> 0.5 mm over 3 s (sudden repositioning) or > 0.3 mm over 15 s (gradual repositioning events) [11]. The respiratory signal was obtained using a band-pass filter of the extracted center-of-mass signal (0.2–0.5 Hz), followed by a moving-average filter (3 s) to remove stochastic noise in the signal.

### Image reconstruction

All PET images were reconstructed using vendor-provided software (JS-Recon12, Siemens, Knoxville, USA), using corrections for both point-spread and time-of-flight using two iterations and 21 subsets. All reconstructions were performed using a voxel-grid of 256 × 256 × 109 indices (2.73 × 2.73 × 2.037 mm) and filtered with a 5-mm Gaussian post-filter.

### Image registration

All PET datasets were co-registered to the CTA images, using anatomical landmarks as described in a previous study [10]. In brief, the images were co-registered based on matching of the blood pool of the two imaging modalities by overlaying the four cardiac chambers for the PET and CTA images. Following PET to CTA co-registration, the PET-PET image registration (PET motion correction) was performed within a sphere (radius = 20 mm) enclosing the aortic valve, thereby, creating ECG-MC and 3 × MC image-sets. The motion correction was performed using nonlinear registrations (daemons) [20]. All motion correction was performed in FusionQuant, thus, employing a post-reconstruction motion correction technique registering the 3D reconstructed frames (FusionQuant, Cedars-Sinai Medical Center) [10].

### Image analysis

**Fig. 2:**
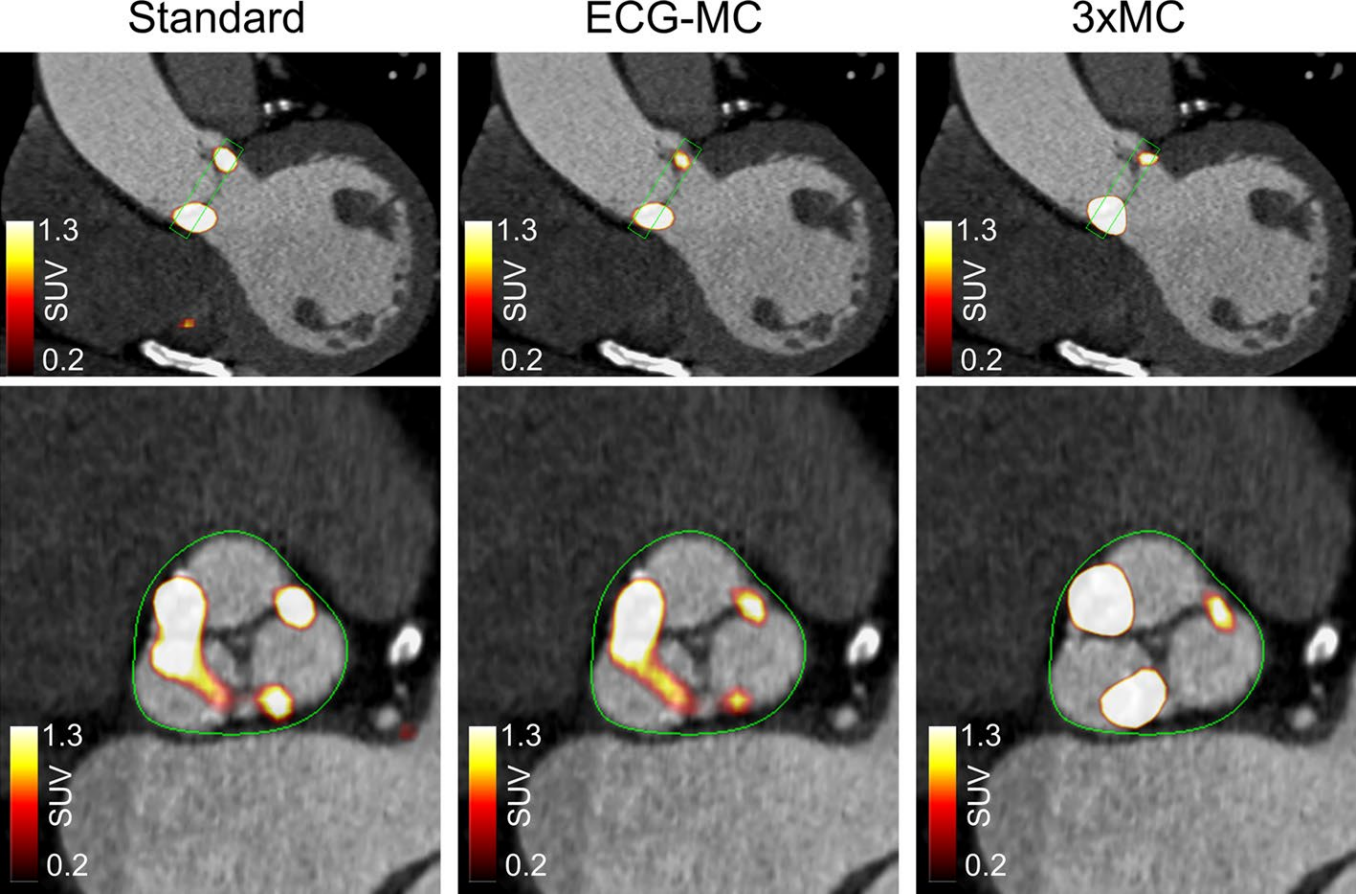
Segmentation of the aortic valve used for quantification of ^18^F-NaF uptake (shown as a green polyhedron). The valve was segmented on the end-diastolic image enclosing on the SUV_max_ observed in the image to ensure high test–retest repeatability. Top row shows the coronal view of the myocardium, while the lower row represents the short-axis view of the aortic valve. ECG-MC = ECG motion-corrected, 3 × MC = triple motion-corrected

*PET Quantification* Using the co-registered PET and CTA images, the valvular uptake was measured using a three-dimensional polyhedron (6-mm thickness) encompassing the valve (Fig. 2). In addition, background blood pool uptake was measured in the center of the right atrium (cylindric volume: radius = 8 mm, height = 9 mm) [12, 16]. Of note, the background blood pool activity was extracted from the right atrium as it offers reduced coefficient of variation in the measurements compared to measurements obtained in the vena cava and brachiocephalic vein, although at the cost of slightly increased blood pool activities [16]. The same volume of interests (VOIs) were used for all three reconstruction protocols. All scans were read in a blinded fashion, with readings of the images 6 weeks apart to reduce the risk of bias in the assessment [10]. All the images were read by a cardiologist certified in echocardiography and computed tomography angiography.

To compensate for variations in the background blood pool activity introduced by variations in the injection-to-scan delays [14], the activity observed in the background at time t (in minutes) after the injection was normalized to a standardized injection-to-scan delay of 60 min using a previously described correction factor [12]:

1 $${\text{SUV}}_{{{\text{Background}}\;{\text{corrected}}}} = {\text{SUV}}_{{{\text{background}}}} \times {\text{e}}^{{ - 0.004 \times \left( {60 - t} \right)}}$$

TBR_max_ measurements were obtained by normalizing the SUV_max_ measured in the segmented valve to the corrected mean standardized uptake value obtained in the right atrium (SUV_Corrected Background_) (Eq. 2).

2 $${\text{TBR}}_{{{\text{max}}}} = \frac{{{\text{SUV}}_{{{\text{max}}}} }}{{{\text{SUV}}_{{{\text{Corrected}}\;{\text{Background}}}} }}$$

To assess the image quality of the resulting reconstructions (Standard, ECG-MC, and 3 × MC), we evaluated the signal -to-noise ratio (SNR) obtained in the background. The SNR was defined as the SUV_max_ obtained in the valve normalized to the standard deviation obtained in the background blood pool (right atrium) [10]. The magnitude of the patient repositioning was measured from the PET-to-PET co-registration using the inverse motion vector fields.

### Statistical analysis

The data were tested for normality using the Shapiro–Wilk test, using corrections for multiple comparisons (Bonferroni corrections). Continuous variables were presented as mean ± standard deviation. Test–retest repeatability of the TBR_max_ assessments was compared using the repeatability coefficient (RC; RC = 1.96*standard deviation with the standard deviation being expressed in % difference from the initial scans. Of note, the standard deviation was calculated from the 14 patients included in this study) and using the Kendall’s Tau measure [12]. Tests for variations in TBR_max_ assessments were evaluated using Pitman–Morgan analyses with *p* values < 0.05 were considered statistically significant.

## Results

All patients underwent two ^18^F-NaF PET/CTA scans within one month. The patients had an average AVCS of 1259 ± 908AU. Of the 14 patients, 11 were acquired with the arms placed above the head, while the remaining three patients were unable to have the scans performed with the arms elevated. Patient demographics are shown in Table 1.

**Table 1:** Patient demographics

Demographics	Value
Age (years)	73 ± 7
Gender (Males)	10 (67.7)
Body mass index (BMI)	27.2 ± 4.3
*Cardiovascular risk factors*	
Diabetes mellitus	4 (26.7)
Current smoker	6 (40.0)
Hypertension	11(73.3)
Hyperlipidemia	10 (67.7)
TIA/CVA	2 (13.3)
Renal disease	0 (0)
CABG	2 (13.3)
PCI	4 (23.7)
*Medications*	
ACE inhibitor	6 (40.0)
ARB	3 (20.0)
Beta blocker	7 (56.6)
Statin	9 (60.0)
*Aortic stenosis grade*	
Mild	7 (47.6)
Moderate	4 (26.7)
Severe	4 (26.7)
Aortic valve calcium score (Agatston)	1269 [246–5774]

Continuous variables reported as mean ± SD or median [range]; categorical variables reported as n (%)

*TIA* transient ischemic attack, *CVA* cerebrovascular accident

### Acquisition and motion estimation

The average dose was 124 ± 6 MBq of ^18^F-NaF. On average, the patients had injection-to-scan delays of 68 ± 11 min (range 59–99 min). During the 30-min acquisitions, patients were found to reposition themselves 3.6 ± 1.3 times (range 1–6). The arm position did not seem to affect the number of repositioning events (Repositioning events: arms up = 4 ± 1, arms down = 4 ± 1). Acquisition individual maximum translations in contrast to the reference frame was of magnitude of 14 mm (3D motion) (3D motion: 14.3 ± 2.3 mm, range = [11.1; 21.8 mm]) (Additional file 1: Supplementary figure 1).

### ***SUV***_***max***_*** and TBR***_***max***_*** assessments***

**Fig. 3:**
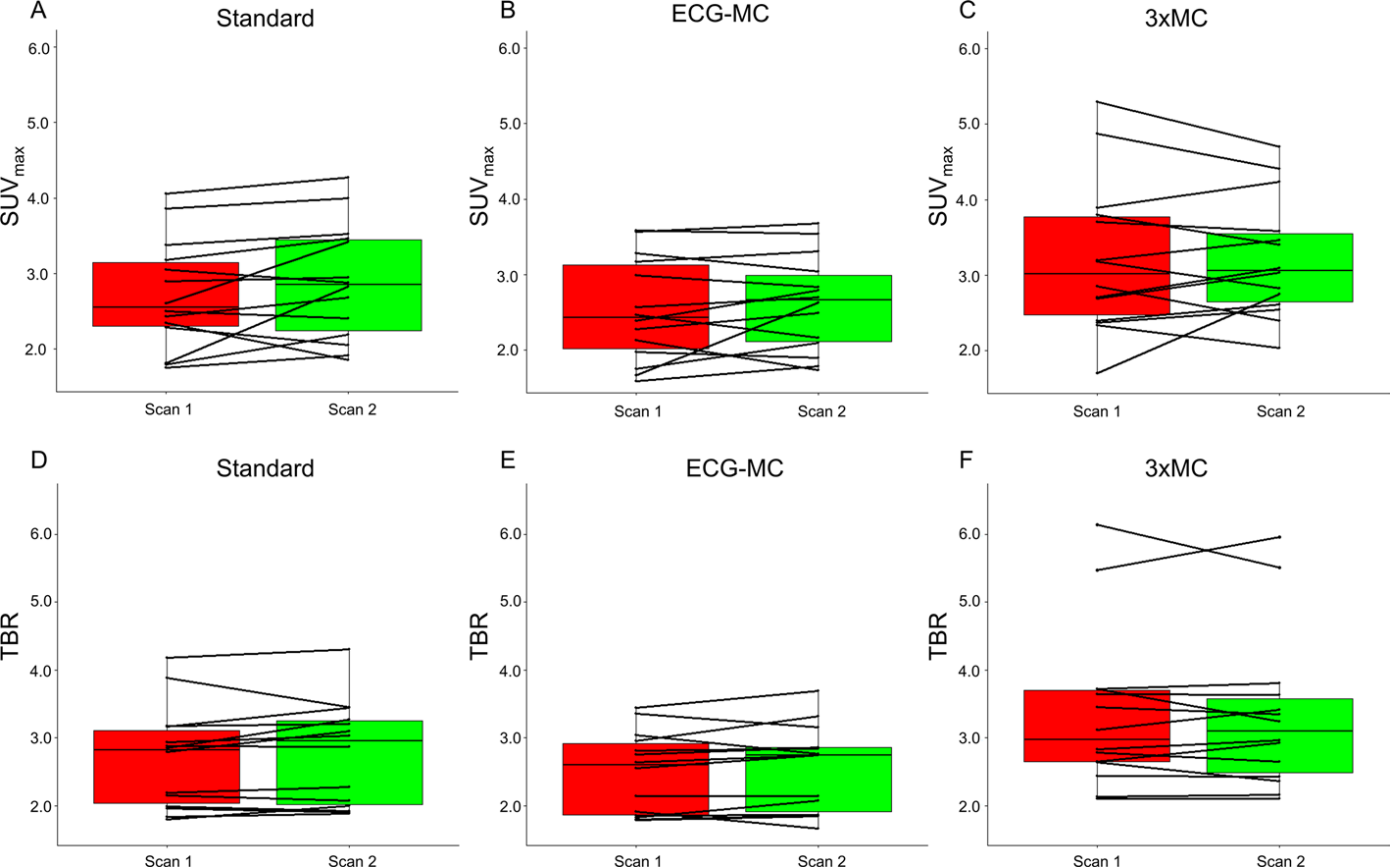
SUV_max_ and TBR_max_ measurements obtained for the three reconstructions. Significantly increased SUV_max_ and TBR_max_ values were observed for the 3 × MC datasets when compared to standard and ECG-MC datasets (Pitman–Morgan analyses, all *p* < 0.05). Standard = end-diastolic imaging, ECG-MC = ECG motion-corrected, 3 × MC = triple motion-corrected. SUV_max_ = maximum standard uptake value, TBR_max_ = maximum target-to-background ratio

**Fig. 4:**
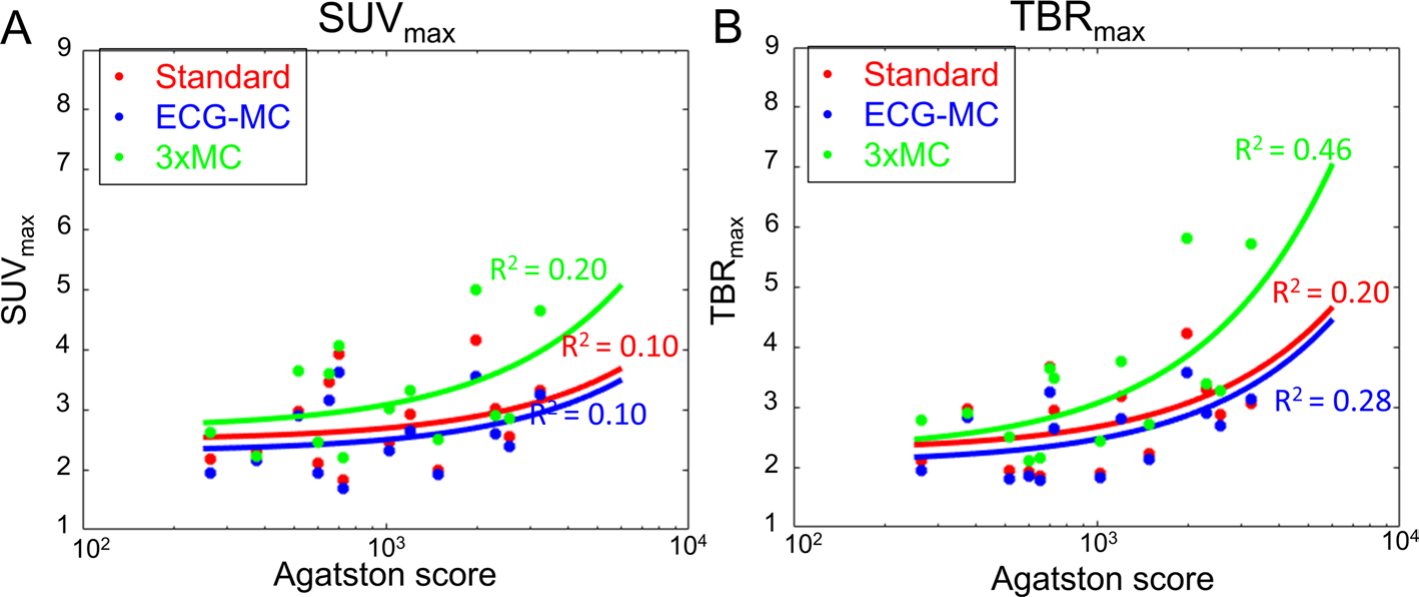
Correlation plots of the aortic valve calcium score and SUV_max_ and TBR_max_. Significantly improved correlations were observed for the 3 × MC when compared to the other reconstructions for both SUV_max_ (**A**) and TBR_max_ (**B**). Of note, the repeated measurements were averaged for the plots. Standard = end-diastolic imaging, ECG-MC = ECG motion-corrected, 3 × MC = triple motion-corrected. SUV_max_ = maximum standard uptake value, TBR_max_ = maximum target-to-background ratio

The 3 × MC reconstruction protocol resulted in increased SUV_max_ and TBR_max_ values when compared to both the standard and ECG-MC protocols (SUV_max_: Standard = 2.8 ± 0.7, ECG-MC = 2.6 ± 0.6, and 3 × MC = 3.3 ± 0.9; TBR_max_: Standard = 2.7 ± 0.7, ECG-MC = 2.5 ± 0.6 and 3 × MC = 3.4 ± 1.2, all *p* values < 0.05) (Fig. 3). Of note, the median variation in the injection-to-scan delay (intra-patient delay) was 6.5 min (inter quartile range = [1.5; 10.5 min]). Correlations plots of the aortic valve calcium score (AVCS) and the SUV_max_ and TBR_max_ (Fig. 4) were improved for the 3 × MC technique when compared to the Standard and ECG-MC techniques (R^2^; SUV_max_: Standard = 0.10, ECG-MC = 0.10, and 3 × MC = 0.20; TBR_max_: Standard = 0.20, ECG-MC = 0.28, and 3 × MC = 0.46).

### Image quality

**Fig. 5:**
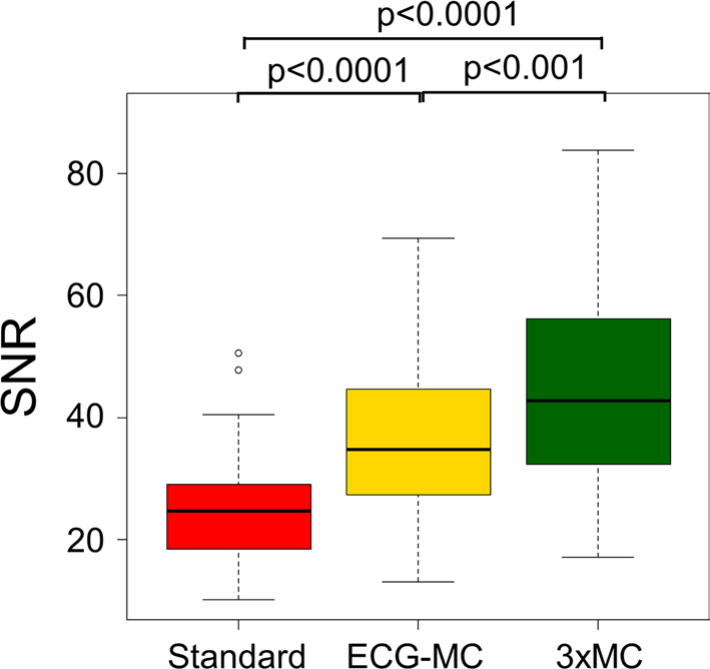
Signal-to-noise ratio (SNR) measurements obtained for the three reconstructions. End-diastolic (Standard) measurements were reported to have significantly lower SNR when compared to the motion-corrected datasets (ECG-MC and 3 × MC). ECG-MC = cardiac contraction motion-corrected, 3 × MC = triple motion-corrected

Lower SNR were obtained for the standard (end-diastolic) image approach (SNR = 25.7 ± 10.1) when compared to the motion-corrected datasets (SNR: ECG-MC = 37.3 ± 12.9, 3 × MC = 45.7 ± 16.9) (both *p* < 0.0001). Noteworthy, the SNR obtained for 3 × MC has improved when compared to the ECG-MC (*p* < 0.001) (Fig. 5).

### Test–retest repeatability measurements

**Fig. 6:**
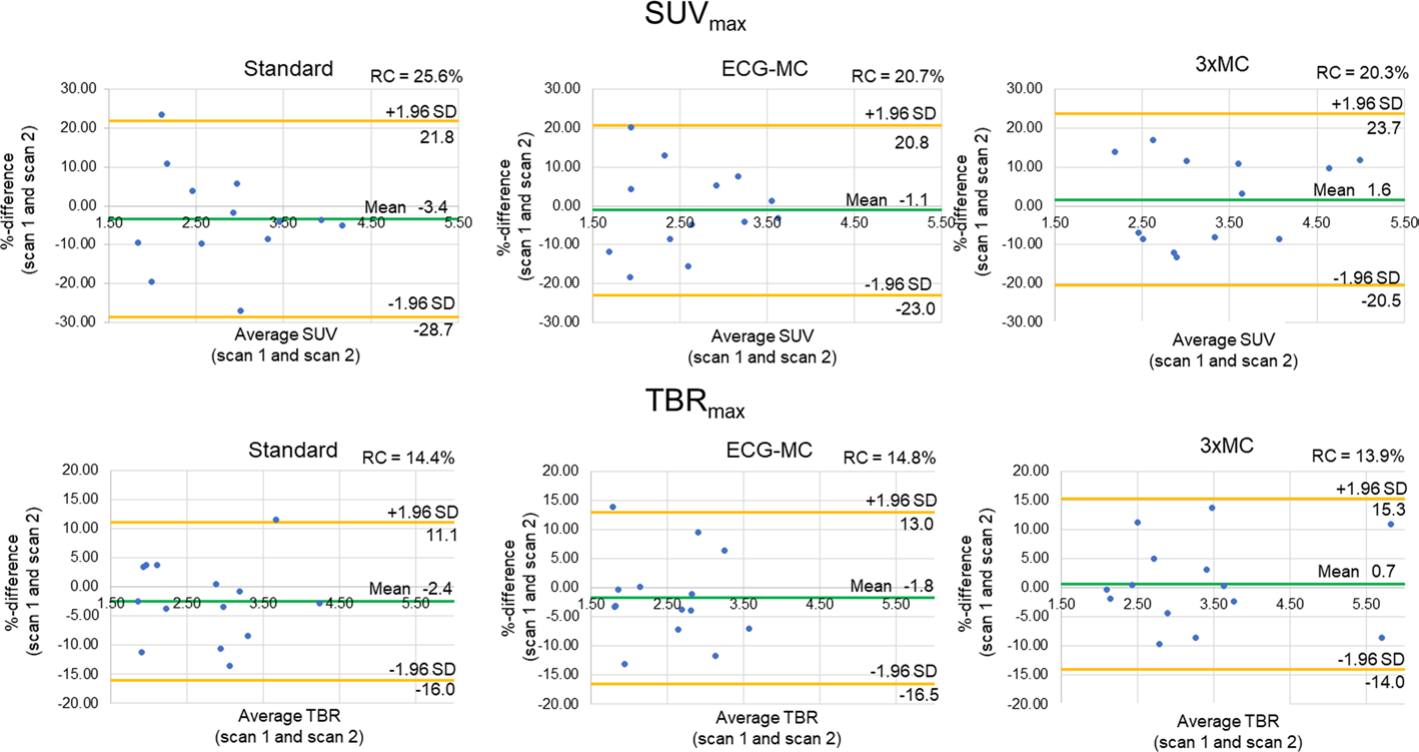
Test–retest repeatability of SUV_max_ and TBR_max_ for standard (end-diastolic), ECG-MC, and 3 × MC reconstruction protocols. RC = repeatability coefficient, TBR_max_ = maximum target-to-background ratio, ECG-MC = cardiac contraction motion-corrected, 3 × MC = triple motion-corrected

**Fig. 7:**
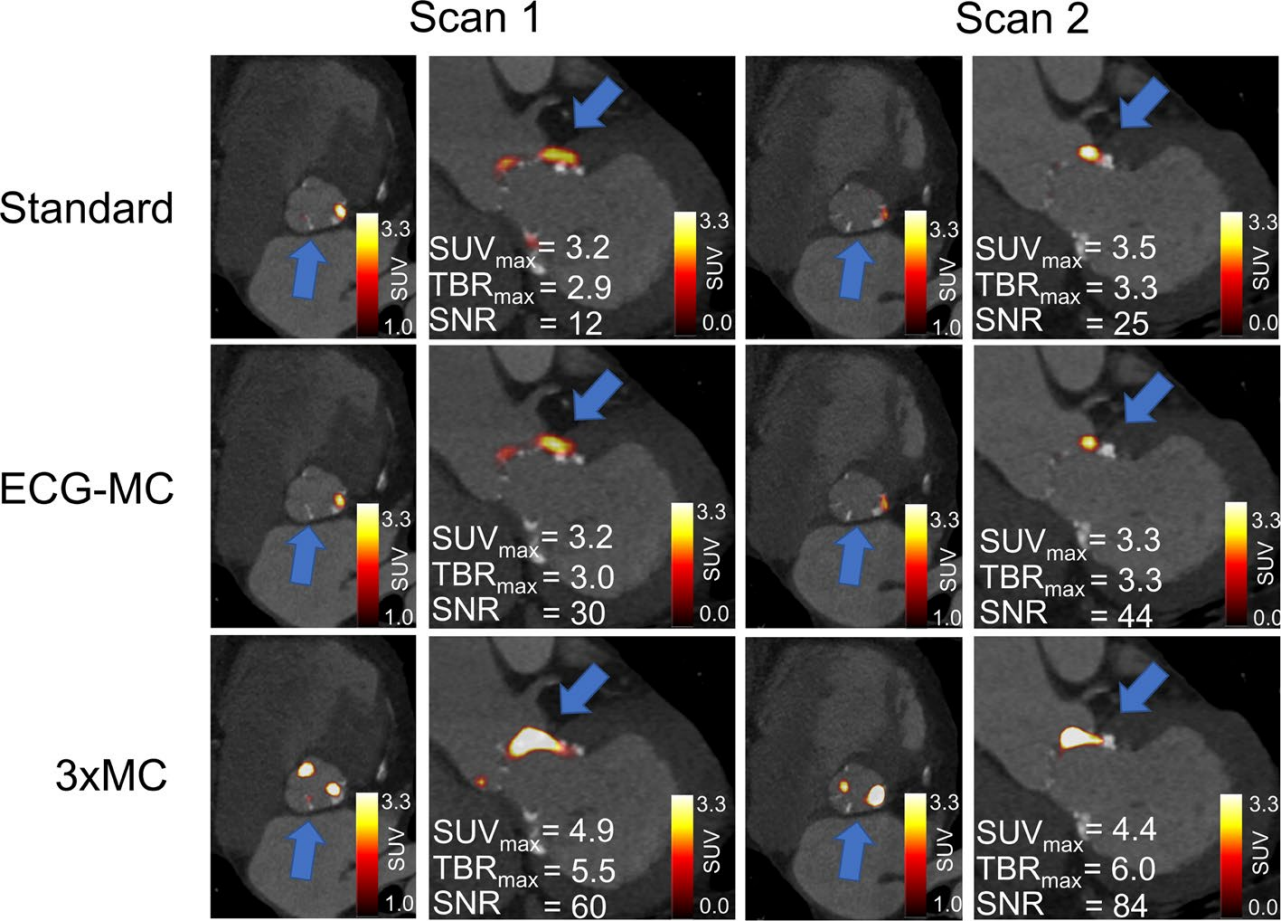
Test–retest valve PET imaging for a patient before and after motion and background blood pool corrections. Standard and ECG-MC images present less specific uptake patterns at the areas with calcification, while 3 × MC images show reproducible uptake patterns for the two scans. ECG-MC = cardiac contraction motion-corrected, 3 × MC = triple motion-corrected, SUV_max_ = maximum standardized uptake value, TBR_max_ = maximum target-to-background ratio, SNR = signal-to-noise ratio

**Fig. 8:**
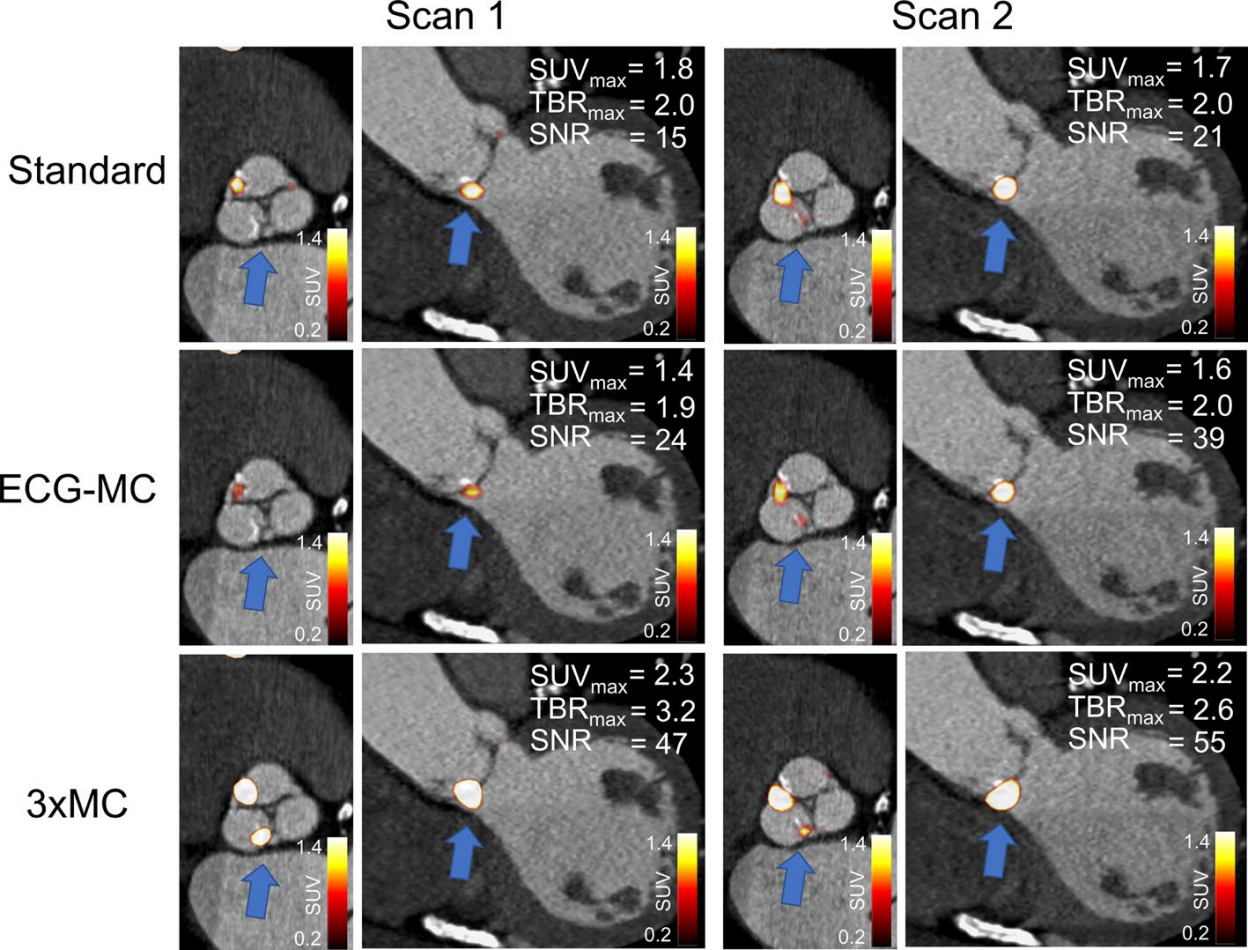
Test–retest valve PET imaging for a patient before and after motion and background blood pool corrections. Standard and ECG-MC images present lower TBR_max_, while 3 × MC images show higher TBR_max_ (68% and 30% increase in scan 1 and scan 2, respectively). ECG-MC = cardiac contraction motion-corrected, 3 × MC = triple motion-corrected, SUV_max_ = maximum standardized uptake value, TBR_max_ = maximum target-to-background ratio, SNR = signal-to-noise ratio

Similar test–retest repeatability coefficient were obtained for all three reconstruction protocols (SUV_max_: Standard = 25.6%, ECG-MC = 20.7%, 3 × MC = 20.3%; TBR_max_: Standard = 14.4%, ECG-MC = 14.8%, 3 × MC = 13.9%, all *p* ≥ 0.79) (Fig. 6). Similarly, the Kendall’s Tau measures showed improved reliability of the 3 × MC reconstruction protocols, although not being statistically significant (SUV_max_: Standard = 0.74, ECG-MC = 0.69, 3 × MC = 0.74; TBR_max_: Standard = 0.80, ECG-MC = 0.78%, 3 × MC = 0.85, all *p* ≥ 0.79). Figures 7 and 8 show two cases of test–retest repeatability of the tracer-uptake before and after motion correction.

## Discussion

We evaluated the use of a novel 3 × MC PET reconstruction technique tested for the first time in PET imaging of aortic valves, including cardiorespiratory and gross patient motion correction. The impact of the 3 × MC protocol was evaluated on five criteria assessing the quantitative and qualitative assessments of the images: SUV_max_, TBR_max_, SNR, the test–retest repeatability, and the correlation between the quantitative measures and AVCS. The 3 × MC was superior to the standard (end-diastolic) and ECG-MC imaging protocols without affecting the test–retest repeatability. The improved localized uptake observed in the aortic valves for the 3 × MC reconstructions correlated better with the AVCS than the standard and ECG-MC protocols, which in combination with the increased SNR might aid the understanding of the pathophysiology in native and bioprosthetic valve diseases.

The assessment of aortic valve microcalcification with ^18^F-NaF can be used to predict aortic valve stenosis progression [1, 4, 16, 21]. Studies to date have established the association between increased TBR_max_ and aortic stenosis progression using either standard (end-diastolic) or ECG-MC image series [1, 10, 15]. However, it is uncertain how much patient and respiratory motion can affect quantitative assessments of ^18^F-NaF uptake (SUV_max_ and TBR_max_). It is therefore of great interest to evaluate potential image improvements offered by the 3 × MC protocol in the context of aortic valve microcalcification. Specifically, the improved co-localization of PET activity with areas of aortic valve calcification is of high interest in studies investigating the various causes of leaflet calcification, i.e., mechanical stress at the leaflet coaptation points versus commissures. Moreover, it might help differentiate between activities originating in the aortic valve from activity from nearby structures (left main stem, mitral valve, left atrium, etc.) by reducing the spillover effect of those structures. This is of importance in cases of bioprosthetic valves, and transcatheter aortic valve implantation (TAVI), where localizing the source of PET activity can help differentiate between bioprosthetic valve leaflet degeneration and remote activity originating from the valve struts or crushed native leaflets in case of TAVI [22]. In this context, partial volume effects strongly affect the activity observed in the aortic valves and their surrounding tissues. It is anticipated that the 3 × MC reconstruction protocol may ameliorate the impact of the apparent partial volume effects because of the co-registration of the gated images with significantly reduced intra-gate motion compared to the standard and ECG-MC reconstruction protocols where several motion patterns blur the resulting images. Therefore, it is believed that the 3 × MC reduces the impact of the partial volume effects while also aiding toward better test–retest repeatability.

In the current study, all patients demonstrated an increase in both SUV_max_ and TBR_max_ when the data were corrected using 3 × MC (cohort-based increase: SUV_max_ = 26%, TBR_max_ = 33%) (Fig. 3), indicating loss of signal when not applying these correction techniques. The improved SUV_max_ and TBR_max_ assessments for the 3 × MC both had preserved repeatability measures and led to a twofold increase in the correlations to the aortic valve calcium score when compared to the Standard and ECG-MC reported results; thus, suggesting that 3 × MC might improve predictions on disease progression (Figs. 3, 4, 6, 7, 8) [1]. Based on these findings, we can conclude that the motion patterns across different patients vary widely, depending on the respiratory translations and patient motion patterns during the acquisitions, which in some cases can introduce variations in the quantitative values exceeding 40% (average increases of the four scans with substantial changes in SUV_max_ and TBR_max_ values: SUV_max_ = 42%, TBR_max_ = 72%). The increase in the correlation scores observed for the TBR_max_ and aortic valve calcium score may translate into lower number of patients required in studies investigating the effects of interventions on ^18^F-NaF PET uptake (as a marker of calcification activity) because any true effect will not be covered by noise within the region of interest.

Motion during the scans has been shown to have a detrimental impact on the image quality, measured as SNR [10, 13]. In concordance with a previous study of coronary plaques, the SNR was significantly reduced for the standard imaging protocol when compared to the motion-corrected protocols [13]. The low SNR observed for the standard protocols is introduced by two arms, low count statistics (25% of the acquired data used for the analyses), and respiratory and patient motion blur embedded in the ECG-gated images [12]. Introducing motion correction (ECG-MC and 3 × MC), the SNR improved as all the data were used in the analyses partly owing to the increased count statistics in the images (100% of the acquired data). The further improvement observed for the 3 × MC was introduced by the corrections for both respiratory and patient motion, which reduced the residual blur introduced to the ECG-gated reconstructions, as shown in Fig. 4. While SNR was significantly reduced using ECG-MC alone compared to 3 × MC, ECG-MC provides greatly improved SNR when compared to the standard assessment and therefore should be considered when 3 × MC is not possible. Of note, the gated reconstructions took 3 min to reconstruct each gate using a 3 year-old PC, introducing a reconstruction time of 48 min per repositioning event.

While some of the discrepancies, in general, may be attributed to changes in the pathological disease, only minor changes in the SUV_max_/TBR_max_ test–retest variability is expected to be introduced by disease progression owing to the slow microcalcification processes which may change the calcification burden by 24% per year (and thus, only 1–2% change in the SUV_max_/TBR_max_ burden should expected) [16]. Therefore, the discrepancy in the impact of the ECG-MC and 3 × MC observed for SNR, TBR_max_, and SUV_max_ indicate that the different motion patterns (cardiac contraction, respiratory and gross patient motion) affect image quality to varying extent. The improved quantitative and qualitative findings observed for the 3 × MC protocol suggest that cardiac contraction is of less importance to correct for in studies of aortic valves when compared to the patient repositioning events and the respiratory translations. Such results were expected as the cardiac contraction affects mainly the coronary arteries, where the right coronary artery has been reported to shift up to 26 mm [23] which is in contrast to the average displacement of the aortic valves (~ 12 mm) [24]. In contrast, respiratory motion has been reported to displace the heart by up to 21 mm [25] and patient repositioning events might shift the heart 5–15 mm [11]. In this context, patient motion (repositioning events) usually happens with low frequency (approximately 3.5 times per scan) and although the translations are few, they introduce non-periodical shifts of the respiratory baseline and, thus, affect the cardiorespiratory motion correction.

Another important finding in our study is the robustness of the data-driven motion detection techniques, as applied to valve imaging studies. A previous study has shown that motion detection techniques employing these assessments are affected by reductions in the count rates [26]. The count rates are mainly affected by two variables: the injected activity and injection-to-scan delay. While the previous study utilizing a 3 × MC protocol (focusing on coronary plaques) had similar injection-to-scan delays as the current valve study (59–99 min) [12], the injection doses, in general, are halved for the studies of aortic valve microcalcification (to 125 MBq) [7, 10, 17], as compared to studies of coronary plaques (250 MBq) [12, 14, 27]).

Given the large dose-reduction in the current study, it was important to test the robustness of the extracted motion patterns (respiratory and patient). In this context, we evaluated the robustness of the technique by evaluating the impact of the data-driven motion detection techniques through assessments of the TBR_max_, SNR, and test–retest repeatability of the TBR_max_ assessments. First, the number of repositioning events reported in this study was in concordance with previous studies [11, 12], suggesting that the robustness of the motion detection is preserved. Motion correction of the detected repositioning and respiratory events introduced an increase in both SUV_max_, TBR_max_, and SNR while preserving a high test–retest repeatability which is in concordance with already established imaging protocols [28]. These findings strongly indicate that the data-driven motion detection technique developed for patient and respiratory motion detection algorithm provides robust and reliable results in valve imaging even when using a low-dose imaging protocol.

### Limitations

In this study, the number of patients was limited to 14 who underwent two PET/CTA scans within a month—this number is limited by the difficulty in obtaining such repeated PET/CT scans with short time interval. Nevertheless, we were able to report substantial improvements for SUV_max_ and TBR_max_ following 3 × MC, which correlated better to the calcium scores. The attenuation correction was not motion-corrected prior to image reconstruction, which might pose another limitation. However, in a previous study from our center, we showed that a respiratory averaged CT attenuation correction did not change the quantitative assessment. Therefore, we do consider this a limitation of this study [29]. Another limitation was the use of only four cardiac and four respiratory gates for each patient repositioning event during the scans; however, double gating imposes count limitations especially in low-dose studies as studied. Further improvement in TBR_max_ values is possible with an increasing number of cardiorespiratory gates or introducing motion correction during the reconstruction, whereby noise in the images will be suppressed.

## Conclusion

3 × MC employed in ^18^F-NaF aortic valve imaging substantially improves the correlation between the obtained aortic valve calcium score and SUV_max_, TBR_max_ and should be considered in PET studies of aortic valves using ^18^F-NaF.

## Supplementary Information

**Additional file 1: Supplementary Figure 1.** Maxmum translations (3D) of the aortic valve obtained for the repeat scans. On average, the aortic valve was translated 14.3 mm during the scans, with a maximum translation of 21.8 mm.

## Data Availability

The data is not publicly available because this study is still ongoing.

## Abbreviations

PET: Positron emission tomography
CTA: Computed tomography angiography
ECG- MC: Cardiac contraction motion-corrected
3 × MC: Triple motion-corrected
TBR_max_: Maximum target-to-background ratio
SUV: Standardized uptake value
VOI: Volume of interest
AVCS: Aortic valve calcification score
