# Supplementary material for: Aortic valve imaging using 18F-sodium fluoride: impact of triple motion correction
Source: EJNMMI Phys. 2022 Jan 29;9:4. doi: 10.1186/s40658-022-00433-7 (PMC8800969; doi:10.1186/s40658-022-00433-7)
Supplement: Supplementary file 1 — Additional file 1: Supplementary Figure 1. Maxmum translations (3D) of the aortic valve obtained for the repeat scans. On average, the aortic valve was translated 14.3 mm during the scans, with a maximum translation of 21.8 mm. [file 40658_2022_433_MOESM1_ESM.docx]

**Supplementary material**

**Aortic valve imaging using ^18^F-Sodium Fluoride: Impact of triple motion correction**

Martin Lyngby Lassen, PhD^a^, Daniele Massera, MD^b^, Evangelos Tzolos, MD^a,c^, Sebastien Cadet, MS^a^, Rong Bing, MD^c^, Jacek Kwiecinski, MD, PhD ^a,d^, Damini Dey, PhD^a^, Daniel S Berman, MD^a^, Marc R Dweck, MD, PhD^c^, David E Newby, MD, PhD^c^, Piotr J Slomka, PhD^a^

^a^Cedars-Sinai Medical Center, Los Angeles, CA, USA

^b^Leon H. Charney Division of Cardiology, New York University School of Medicine, New York, NY, USA

^c^British Heart Foundation Centre for Cardiovascular Science, Clinical Research Imaging Centre, Edinburgh Heart Centre, University of Edinburgh, Edinburgh, United Kingdom

^d^Department of Interventional Cardiology and Angiology, Institute of Cardiology, Warsaw, Poland

**Corresponding author:** Piotr Slomka, Artificial Intelligence in Medicine Program, Cedars-Sinai Medical Center, 8700 Beverly Boulevard, Metro 203, Los Angeles, CA 90048, USA. Tel.: 310-423-4348, Fax: 310-423-0173.

**Supplementary Figure 1**

Maxmum translations (3D) of the aortic valve obtained for the repeat scans. On average, the aortic valve was translated 14.3mm during the scans, with a maximum translation of 21.8mm.

**
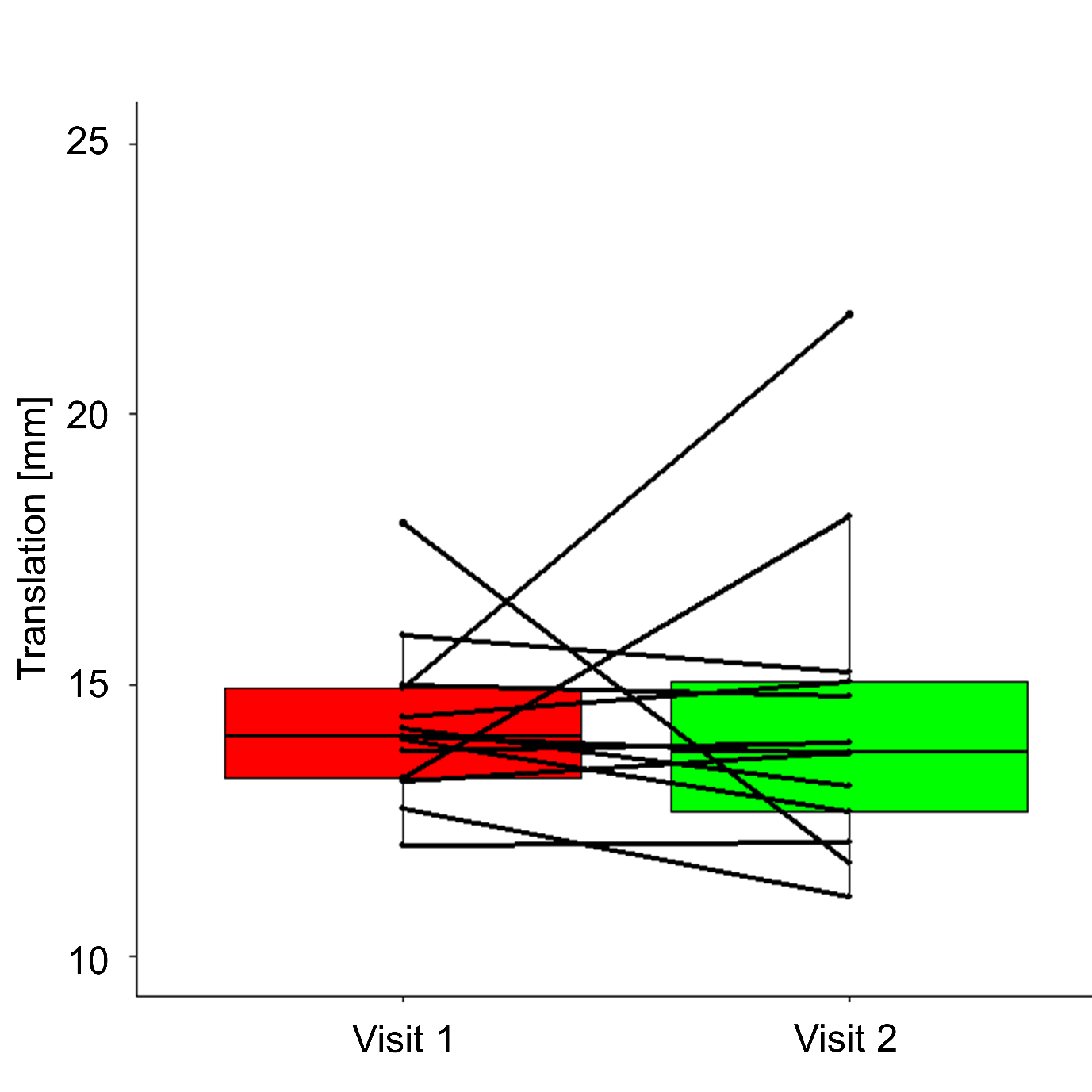
**
